# Comparative Analysis of *Adelphocoris suturalis* Jakovlev (Hemiptera: Miridae) Immune Responses to Fungal and Bacterial Pathogens

**DOI:** 10.3389/fphys.2021.646721

**Published:** 2021-03-18

**Authors:** Meiqi Ma, Libin Guo, Chengjie Tu, Aoli Wang, Letian Xu, Jing Luo

**Affiliations:** State Key Laboratory of Biocatalysis and Enzyme Engineering, School of Life Sciences, Hubei University, Wuhan, China

**Keywords:** *Adelphocoris suturalis* Jakovlev, immune system, RNA-seq, *Beauveria bassiana*, *Enterobactor cloacae*

## Abstract

The wide-spread culture of transgenic Bt cotton resisting the infamous cotton bollworms has reduced the adoption of broad-spectrum insecticides to a large extent. Consequently, the non-targeted insect *Adelphocoris suturalis* Jakovlev has become a major cotton pest in China. Entomopathogenic microbes show promising results for controlling this pest in the future, but *A. suturalis* innate immune responses to these pathogens are poorly understood. Here, we used the entomopathogenic fungus *Beauveria bassiana* and the Gram-negative pathogenic bacteria *Enterobactor cloacae* to infect *A. suturalis* nymphs, followed by high throughput RNA-seq to analyze the immune transcriptomes of *A. suturalis* in response to the two pathogens. A total of 150 immunity-related genes were identified, including pattern recognition receptors, extracellular signal modulators, signal pathways (Toll, IMD, JNK, and JAK/STAT), and response effectors. Further quantitative real-time PCR analysis demonstrated that *B. bassiana* and *E. cloacae* were recognized by different receptors (GNBP and PGRP, respectively); activated Toll pathway and IMD pathway respectively; and both induced expression of the effector gene Defensin. However, melanization is suppressed in *B. bassiana*-infected nymphs. Collectively, this study provides a transcriptomic snapshot of the *A. suturalis* immune system, and at the genetic level, gains multifaceted insights of the immune response to fungal and Gram-negative bacterial pathogens. Ultimately this work pioneers the study of molecular mechanisms underlying immune interactions between *A. suturalis* and its pathogens and assists in the development of novel mitigation strategies to control this pest.

## Introduction

Over the past two decades in China, transgenic cottons expressing *Bacillus thuringiensis* (Bt) toxins have been widely cultured to resist the refractory cotton bollworm *Helicoverpa armigera* (Wu et al., 2008). This wide-spread control measure substantially reduced the adoption of broad-spectrum insecticides, decreasing selection pressure over Bt-insensitive mirid bug species (Hemiptera: Miridae) (Lu and Wu, 2011). Consequently, one member of mirid bug species, *Adelphocoris suturalis* Jakovlev, has now become a major cotton pest in China (Li et al., 2010). Biological control methods including transgenic plants, entomopathogenic microbes, or the combination of both, showed promising efficacy in pest’s management (Yajuan and Wu, 2010; Luo et al., 2017b). Many studies have focused on *A. suturalis* ecology and physiology (Lu et al., 2011; Zhang et al., 2011; Feng et al., 2012; Luo et al., 2017a), reporting its transcriptomes at different developmental stages and pheromone biosynthesis-related genes (Luo et al., 2014; Tian et al., 2015). Together these reports identified candidate genes for further transgenic plants development. However, the immune responses regarding the interactions between *A. suturalis* and microbial pathogens, remain poorly understood.

*Beauveria bassiana* is an entomopathogenic fungus, extensively used as a commercial biopesticide, and which has been proven to competently kill a broad range of pests including Miridae (Boomsma et al., 2014; Mascarin and Jaronski, 2016; Portilla et al., 2019). The infection process starts when a sticky conidium of *B. bassiana* adheres to the host cuticle, germinates to form a germ tube and an appressorium, and subsequently the cuticle is penetrated by mechanical pressure and hydrolysis of secreted enzymes (Mascarin and Jaronski, 2016). After overcoming the host physical barriers, the hyphae can invade the insect hemocoel, where blastospores proliferate, secreting toxins and depriving the host of essential nutrients, eventually leading to the death of the insects (Boomsma et al., 2014; Valero-Jimenez et al., 2016; Wang and Wang, 2017). Gram-negative bacteria *Enterobactor cloacae* is a common nosocomial pathogen that can also infect insects and trigger immune responses (Zou et al., 2008; Xiong et al., 2015; Yang et al., 2017). In addition to the potential application in pest management, these two pathogens are also widely used models that comprehensively study insect immune responses (Zou et al., 2010).

In insects, the innate immune system, consisting of physical barriers, humoral, and cellular immune responses, is central to defending against parasitic or pathogenic infections (Lemaitre and Hoffmann, 2007). The physical barrier is mainly attributed to the cuticle (Moret and Moreau, 2012). Successful pathogen invasion activates humoral and cellular innate immune responses. Generally, cellular immunity includes phagocytosis, encapsulation, and nodulation by hemocytes in the hemolymph (Fauvarque and Williams, 2011). Humoral responses result from the production of immune effector molecules and melanization, elicited by the recognition of pathogen associated molecular patterns (PAMPs) by pattern recognition receptors (PRRs) such as peptidoglycan recognition protein (PGRP), Gram-negative binding protein (GNBP), and C-type-lectin (CTL) (Leulier et al., 2003; Gottar et al., 2006). Signal modulation and signal transduction including Toll, IMD, JNK, and JAK/STAT pathways are then subsequently initiated, leading to the generation of effector molecules such as attacin, defensin, and lysozyme (Ferrandon et al., 2007). Where fungal pathogens and Gram-positive bacteria are responsible for the Toll pathway, and Gram-negative bacteria can activate the IMD pathway (Lemaitre et al., 1995, 1996; Michel et al., 2001). The pathogen recognition can also trigger melanization, through activation of prophenoloxidase (PPO), melanin synthesis and pathogen sequestration (Cerenius and Söderhäll, 2004; Cerenius et al., 2008). The insights into immune responses of *A. suturalis* against different pathogens could improve our understanding of its immune system and guide the development of novel control methods.

In this work, we first investigated the survival rates of *A. suturalis* injected with *B. bassiana* and *E. cloacae* to confirm susceptibility. We then utilized high throughput RNA-seq to analyze the *A. suturalis* immune transcriptome in response to these two pathogens. The resulting sequence information was mapped to other insect immune-related datasets to identify putative immunity-genes in *A. suturalis*. Expression patterns of 17 genes from RNA-seq results were further validated by quantitative real-time PCR. This study provided a transcriptomic snapshot of the *A. suturalis* immune system and gained multifaceted insights of the immune response to fungal and Gram-negative bacterial pathogens at the genetic level. Ultimately this work will pioneer the study of molecular mechanisms underlying immune interactions between *A. suturalis* and its pathogens and assist in the development of novel mitigation strategies to control this pest.

## Materials and Methods

### Insect Rearing and Pathogenic Infection Bioassays

*Adelphocoris suturalis* nymphs were obtained from Huazhong Agricultural University and reared with fresh mung bean seedlings and pea aphids (*Acyrthosiphon pisum*) in clean plastic containers at 25 ± 2°C, 70 ± 5% relative humidity, under the photoperiod of 16 h light/8 h dark (Luo et al., 2017a). *E. cloacae* (strain no.1.2022) was obtained from China General Microbiological Culture Collection Center and was cultured in LB (Luria-Bertani) medium at 37°C. *B. bassiana* isolate 093 was cultured on Potato Dextrose Agar (PDA) medium at 25°C. As a dispersing agent, 0.05% (v/v) Tween 80 solution was used to collect the fungal conidia. In total, 150 newly molted third-instar nymphs were randomly selected, divided into three parts, and injected with the 0.05% Tween solution (as the control group) or *E. cloacae* bacterial Tween suspension (about 1.9 × 10^8^ cfu mL^–1^), or *B. bassiana* conidial Tween suspension (about 1 × 10^7^ conidia mL^–1^), respectively (Xiong et al., 2015). Each nymph was injected with 20 nL in volume, *via* a microinjection, using a microinjector (World Precision Instruments, Sarasota, FL, United States). Before the injection, the nymphs were starved for 2 h, and the conjunctives between the metathorax and the first abdominal segment comprised the injection site (Liu et al., 2019). A more detailed process of the microinjection is described in Liu et al. (2019). Survival was counted every 6 h.

### cDNA Library Preparation and Illumina Sequencing

To obtain immune transcriptomes with large read coverage, we selected two time points to sample, lethal time of 10% (LT10) and 50% (LT50) (Xiong et al., 2015; Zhang et al., 2017; Xu et al., 2018). Due to the different pathogenicity and lethality dynamics, *E. cloacae-*infected samples were collected 49 and 84 h post infection. The Tween treatment group was sampled at 49 h after injection, and three live but weak nymphs in each group were pooled as one sample and a whole body of a nymph was sampled here, the total RNA of three biological replications of each treatment were extracted, respectively, so as to extract RNA as much as possible. Total RNA was extracted with an SV total RNA isolation system (Promega, Madison, WI, United States). Then, the mixed RNA of three biological replications of each treatment was used for RNA-seq (Zhang et al., 2015). Five cDNA libraries (Control, Ec_6h, Ec_12h, Bb_49h, and Bb_84h) were constructed using 3 μg of RNA and sequenced on an Illumina Hiseq^TM^ 2000 system (Illumina, United States). FASTQ files of raw reads were produced and sorted by barcodes for subsequent analysis. Raw reads were submitted at the NCBI in the SRA database (PRJNA692314).

### Sequencing Assembly, Annotation and Gene Expression Analysis

Adaptor sequences, low-quality reads (Quality score < 20), and contaminated reads were removed to obtain clean data. The percentage of Q20 data were 98.93% (Control), 98.95% (Ec_6h), 98.91% (Ec_12h), 98.78% (Bb_49h), and 98.81% (Bb_84h). The clean reads were then *de novo* assembled to yield contigs using the Trinity software (Grabherr et al., 2011). Transcript sets were clustered to reduce sequence redundancy and to generate unigenes with CD-HIT software (Li and Godzik, 2006; Fu et al., 2012).

All unigenes were annotated against the non-redundant (NR) sequence database and Swiss-prot database using BLASTX (*E*-value < 1e^–5^). Gene ontology (GO) annotations for all unigenes were obtained to predict gene functions through the Blast2GO program against the GO database (Ashburner et al., 2000; Conesa et al., 2005). Pathway annotation was performed *via* the online KAAS tool at the Kyoto Encyclopedia of Genes and Genomes (KEGG) (Kanehisa and Goto, 2000).

The RSEM package was utilized to calculate normalized gene expression values, fragments per kilobase per million mapped reads (FPKM) (Trapnell et al., 2010; Li and Dewey, 2011). A differentially expressed gene (DEG) analysis was performed based on the significance level using the R package, DEGseq (*p*-adjust < 0.001, fold change > 2) (Wang et al., 2010).

### Identification of Immunity-Related Genes

Amino acid sequences of known immunity-related genes from other insect species were used as templates for a BLASTX search of *A. suturalis* unigenes. The immune-related gene database was downloaded from the orthodb database^1^ and mapped insect species included *Drosophila melanogaster*, *Anopheles gambiae*, *Apis mellifera*, *Bombyx mori*, *Tribolium castaneum*, and so on (Xiong et al., 2015; Zhang et al., 2015). The potential *A. suturalis* immunity-related genes were identified manually through predictions of similar structure domains against matched genes. The conserved domains were detected using the CD-search tool^2^.

### Reverse Transcription-Quantitative PCR Analysis

Reverse transcription-quantitative PCR (RT-qPCR) analysis was adopted to validate immunity-related gene expression patterns from RNA-seq results. RNA samples were prepared using the same method we described above. A total of 2 μg of RNA was reverse transcribed using PrimeScript^TM^ RT Master Mix (Takara, Shiga, Japan) and RT-qPCR was performed on an ABI 7300 Real-Time PCR System (United States) using GoTaq RT-qPCR Master Mix (Promega, Madison, WI, United States). All samples obtained at different timepoints have corresponding Tween controls. Three biological replications were conducted for each treatment. Four technical replications were done for each sample in RT-qPCR. PCR conditions consisted of 94°C for 5 s, followed by 40 cycles of 59°C for 20 s and 72°C for 20 s. The primers used are described in Supplementary Table 1. The *RPS15* gene was used as the reference gene (Luo et al., 2018; Lü et al., 2018). Data were collected, exported to EXCEL, and analyzed by the 2^–ΔΔ*Ct*^ method (Schmittgen and Livak, 2008). The mean Ct value of four technical replications in each sample was used for the gene expression analysis, and the relative expression level of each treatment was the average of the three biological replications.

## Results

### Survival Analysis of *Adelphocoris suturalis* Nymphs Infected With *Beauveria bassiana* and *Enterobactor cloacae*

**FIGURE 1:**
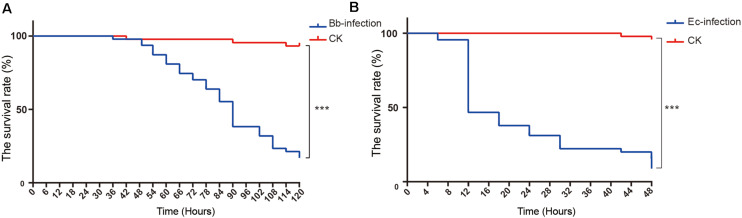
The survival curves and transcriptomic changes of *A. suturalis* nymphs injected with *B. bassiana*, *E. cloacae* or mock (Tween-80). **(A)** The survival curve of *B. bassiana*-infected nymphs; **(B)** The survival curve of *E. cloacae*-infected nymphs. The log-rank test was used to evaluate the significance of differences between groups (***p < 0.001).

The survival curves of nymphs infected by *E. cloacae* and *B. bassiana* were significantly lower than that of the mock control (nymphs injected with Tween 80, Figures 1A,B, Log-rank test, *p* < 0.001), demonstrating that *A. suturalis* is very sensitive to these two pathogens. The LT50 of nymphs infected by *E. cloacae* was 12 h, and 84 h for nymphs infected by *B. bassiana*, indicating that *E. cloacae* has a more efficient lethality to *A. suturalis* than *B. bassiana.*

### Identification and Functional Classification of DEGs of *Adelphocoris suturalis* in Response to *Beauveria bassiana* and *Enterobactor cloacae* Infection

**FIGURE 2:**
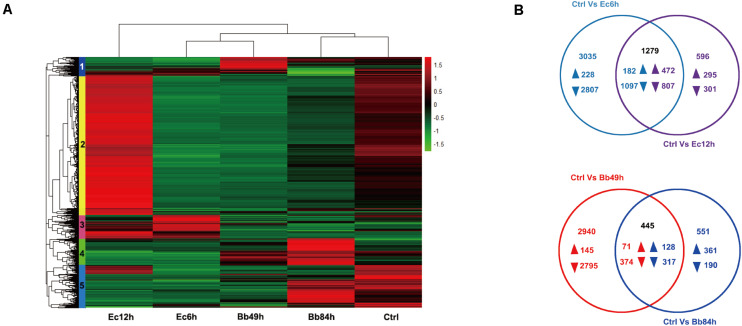
Transcriptome analysis of *A. suturalis* nymphs in response to *B. bassiana* and *E. cloacae* infection. **(A)** Hierarchical clustering analysis of DEGs in the *A. suturalis* nymphs infected with *B. bassiana*, *E. cloacae*, and Tween 80 (*p* < 0.001, fold change > 2). The heatmap is divided into five clusters that are marked by different color boxes on the left; **(B)** Venn diagrams of DEGs in *B. bassiana*- or *E. cloacae*- challenged *A. suturalis* nymphs. The overlapping regions show genes that are regulated in both samples. The non-overlapping regions represent genes that are specifically regulated in corresponding sample. The directions of transcript level changes are indicated by upward- and downward- pointing arrows.

A total of 29,142 unigenes were annotated based on BLASTX searches, and 5,581 DEGs from the five libraries were obtained according to DEGseq results. Hierarchical clustering of DETs identified five clusters that represented differential expression patterns (Figure 2A and Supplementary Table 2). One-hundred-and-twenty-nine genes in cluster 1 were upregulated at 49 h after *B. bassiana* infection. Transcript levels of 2,776 genes in cluster 2 genes were declined at 6 h but 542 genes in this cluster increased at 12 h post *E. cloacae* infection. Cluster 3 (553 genes) showed 380 genes increased 6 h after bacterial infection (208 genes increased and 25 genes decreased at 12 h), while 401 genes in cluster 4 showed the gene upregulated 84 h post fungal infection.

**FIGURE 3:**
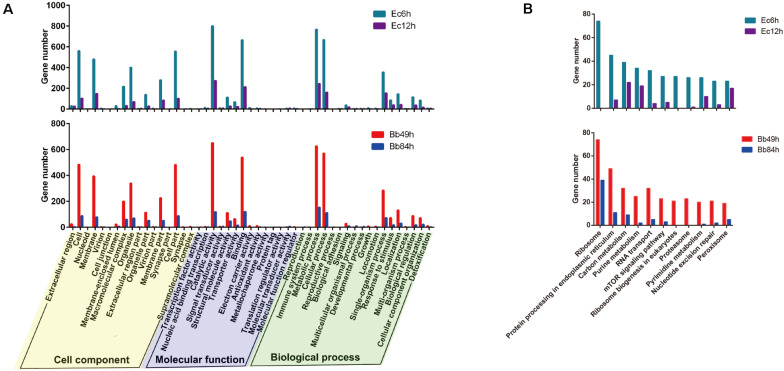
Go and KEGG analysis of DEGs. **(A)** Go (Gene ontology) enrichment and **(B)** KEGG pathway enrichment analysis of DEGs in the *A. suturalis* transcriptomes. Level 2 GO assignments are made in terms of cellular components, molecular functions, and biological processes.

According to the Venn diagram analysis of the DEGs, in the *B. bassiana-*infected nymphs, 1,251 genes were specifically regulated in 49 h (145 up- and 2,795 down-regulated), and 551 genes were exclusively regulated in 84 h (361 up- and 190 down-regulated). In the *E. cloacae-*infected nymphs, 3,035 genes were specifically regulated 6 h post-infection (228 up- and 2,807 down-regulated), and 596 genes were exclusively regulated 12 h post-infection (295 up- and 301 down-regulated) (Figure 2B). Based on the GO enrichment analysis, most DEGs in the *B. bassiana-* and *E. cloacae-*challenged nymphs were enriched in catalytic activity, binding, metabolic and cellular processes, and in cell parts (Figure 3A). The KEGG pathway analysis indicated that most DEGs of bacteria-infected nymphs were enriched in ribosome, protein processing in endoplasmic reticulum, carbon metabolism, and purine metabolism (Figure 3B and Supplementary Table 4), and for fungi-infected nymphs, most DEGs were enriched in protein processing in endoplasmic reticulum, RNA transport, and carbon metabolism (Figure 3B and Supplementary Table 4).

### Immunity-Related Genes of *Adelphocoris suturalis* in Response to Two Pathogens

**FIGURE 4:**
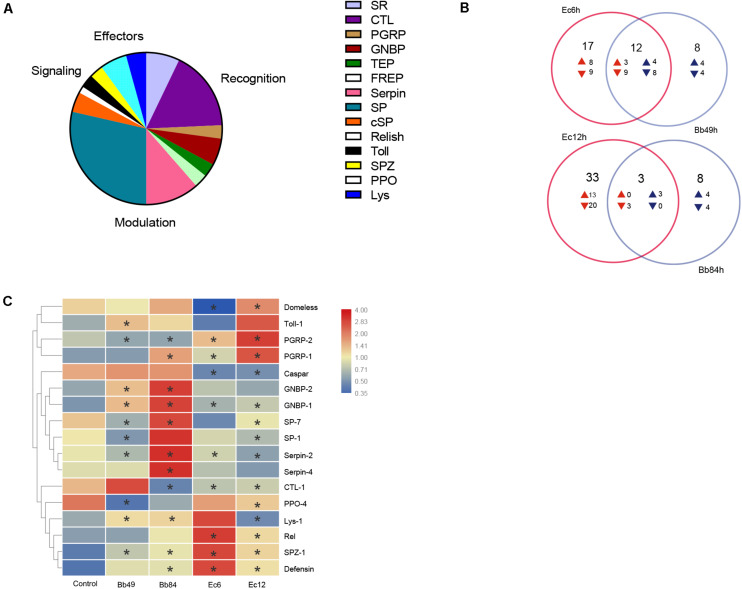
*A. suturalis* immunity-related genes analysis. **(A)** Distribution of *A. suturalis* immunity-related genes in categories of recognition, signaling, regulation, and effectors; **(B)** Venn diagrams of immunity-related DEGs show that fungal and bacterial infections can regulate different immune-related genes. Overlapping regions show genes that regulated in both samples, while non-overlapping regions represent genes that are specifically regulated in corresponding sample. The directions of transcript level changes are indicated by upward- and downward- pointing arrows; and **(C)** Quantitative real-time PCR analysis of the *A. suturalis* immunity-related gene expression, the *A. suturalis* ribosomal protein 15S gene (*RPS15*) was used as an internal control. Data are normalized to the expression level in Tween 80-treated nymphs (*p < 0.05).

A total of 150 immunity-related genes were identified (Figure 4A and Supplementary Table 3) and classified as: pathogen recognition, extracellular signal modulation, intracellular signal transduction (Toll, IMD, JNK, and JAK/STAT pathways), immune response effectors, and others. Thirty-four pathogen recognition receptors were identified, including five scavenger receptors (*SR*), 12 C-type lectins (*CTL*), three galectins (*GALE*), two *PGRP*, four *GNBP*, two Thioester-containing proteins (*TEP*), two *FREP* genes, two nimrods, and two *DRPR* (Draper) genes. Eight serine protease inhibitors (*Serpin*), 20 serine proteases (*SP*) and three clip domain serine proteases (*cSP*) involved in signal modulation were identified. One Relish, one Caspar, one Cactus, two Toll, and two spätzle (*SPZ*) signal transduction molecules were identified in the transcriptome. As for immune response effectors, four prophenoloxidases (*PPO*) of melanization, three lysozymes (*Lys*), one lysozyme-like protein (*LLP*), and one Defensin (*DEF*) were identified according to the transcriptome analysis. The Venn diagrams of immunity-related DEGs show that fungal and bacterial infections can regulate different immune-related genes (Figure 4B).

### Validation of Immunity-Related Genes Expression Patterns by RT-qPCR

For *B. bassiana-*infected nymphs at 49 h or 84 h, genes in recognition receptors (*GNBP1* and *GNBP2*), signal modulation (*Serpin2, Serpin4*, and *SPZ*), Toll pathway (*Toll-1*), and effectors (*Lys1* and *Defensin*) were significantly up-regulated, while *PGRP2* in recognition receptors and *PPO4* in melanization were down-regulated. Recognition receptor genes, *SP1* and *SP7*, were downregulated at 49 h post infection and then upregulated at 84 h, while *CTL1* showed the opposite pattern (Figure 4C).

For *E. cloacae-*infected nymphs, *PGRP1, PGRP2, SPZ1*, and *Defensin* were significantly upregulated. *CTL1, GNBP1, Serpin2, Serpin4, SP1*, and *PPO4* were down-regulated. Similarly, the *Domeless* signal pathway was downregulated at 6 h post infection and upregulated at 12 h, while *Lys1* showed a reverse pattern (Figure 4C).

## Discussion

In this study, we used the entomopathogenic fungus *B. bassiana* and the pathogenic bacteria *E. cloacae* to infect *A. suturalis* nymphs. Great sensitivity to these two pathogens with high mortality rates was reported (Figure 1). With high-throughput RNA-seq and *de novo* assembly in the absence of a reference genome, we matched the pathogen-challenged *A. suturalis* transcriptomic information with other insects’ immune genes, to identify immunity-related genes; and validated expression patterns of some immune genes by RT-qPCR (Supplementary Table 3 and Figure 4C). Altogether this provides a comprehensive insight of the *A. suturalis*’ immune responses to fungal and bacterial pathogens.

Based solely on transcriptomic information from mixed samples, both *B. bassiana* and *E. cloacae* sharply evoked the expression of *A. suturalis* immunity-related genes (Supplementary Table 3). However, *B. bassiana* and *E. cloacae* infection triggered differential expression patterns (Figures 4B,C), providing multifaceted information about the *A. suturalis* immune system. Where for *B. bassiana*-challenged nymphs, GNBP recognition receptors (*GNBP1* and *GNBP2*) were significantly up-regulated, while PGRP receptors (*PGRP1* and *PGRP2*) were remarkably up-regulated in *E. cloacae*-infected nymphs (Figure 4C), indicating that different receptors are involved in the recognition of circulating fungal and Gram-negative bacterial pathogens, which is analogous to *Drosophila* (Lemaitre et al., 1997; Gottar et al., 2002, 2006). *SPZ* (Spätzle, *SPZ1*) and Toll (Toll-1) (Lemaitre et al., 1996) in *B. bassiana*-infected nymphs were up-regulated (Figure 4C), indicating that this fungus activated the Toll pathway of *A. suturalis*. Caspar is a suppressor of antibacterial immunity and Relish is a transcription factor of the IMD pathway (Hedengren et al., 1999; Kim et al., 2006). The down-regulation of Caspar and the up-regulation of Relish in *E. cloacae*-infected nymphs indicated that the IMD pathway was triggered (Figure 4C). Due to the fact that third-instar nymphs need almost 4 days to develop to fourth-instar nymphs, all the sampled nymphs were still at the third-instar. Theoretically, the nymphs within a same instar, which reared in the same condition with the same treatment, may have similar gene expression patterns. Thus, we chose only one control (Crtl_49h) in the transcriptomic analysis for concision of Figure 2A. Whether the control nymphs at different timepoints have similar gene expression patterns, however, needs to be studied further before being confirmed.

The activation of the Toll and IMD pathways by fungal and gram-negative bacterial infections, respectively, is congruent with previous reports in other insects, such as *Drosophila melanogaster* (Ferrandon et al., 2007). Defensin, an antimicrobial peptide (AMP) (Richman et al., 1996), was upregulated following infection with both pathogens. Previous work suggested that Defensin is required for the host defense against gram-positive bacteria, but is dispensable against invading Gram-negative bacteria or fungi (Blandin et al., 2002; Ferrandon et al., 2007). We speculate that the *B. bassiana* and *E. cloacae* infection may give rise to subsequent opportunistic bacterial infection, but further studies are needed to prove this hypothesis. *PPO4* of melanization was down-regulated in *B. bassiana-*challenged nymphs and up-regulated in *E. cloacae*-infected nymphs. We speculate that toxins secreted by *B. bassiana* may restrain the melanization, but again, in-depth studies are required to support such a hypothesis.

Overall, this work provides a comprehensive view of *A. suturalis*’ immune responses to fungal and bacterial infections at the genetic level and will unravel the molecular mechanisms underlying immune interactions between *A. suturalis* and pathogens. Furthermore, this study will guide the development of efficacious entomopathogenic agents to control this pest.

## Data Availability Statement

The datasets presented in this study can be found in online repositories. The names of the repository/repositories and accession number(s) can be found below: NCBI SRA; BioProject ID PRJNA692314 and BioSample accession SAMN17319809.

## Author Contributions

LX and JL: conceptualization and resources. LX: methodology and supervision. MM: software, formal analysis, investigation, data curation, writing original draft preparation, and visualization. MM, LG, CT, and AW: validation. MM and LG: writing review and editing. JL: project administration and funding acquisition. All authors have read and agreed to the published version of the manuscript.

## Conflict of Interest

The authors declare that the research was conducted in the absence of any commercial or financial relationships that could be construed as a potential conflict of interest.

## Footnotes

1 http://cegg.unige.ch/orthodb7

2 https://www.ncbi.nlm.nih.gov/Structure/cdd/wrpsb.cgi

## References

[B1] Ashburner M. Ball C. A. Blake J. A. Botstein D. Butler H. Cherry J. M. (2000). Gene ontology: tool for the unification of biology. the gene ontology consortium. *Nat. Genet.* 25 25–29. 10.1038/75556 10802651 PMC3037419

[B2] Blandin S. Moita L. F. Köcher T. Wilm M. Kafatos F. C. Levashina E. A. (2002). Reverse genetics in the mosquito *Anopheles gambiae*: targeted disruption of the defensin gene. *EMBO Rep.* 3 852–856. 12189180 10.1093/embo-reports/kvf180 PMC1084233

[B3] Boomsma J. J. Jensen A. B. Meyling N. V. Eilenberg J. (2014). Evolutionary interaction networks of insect pathogenic fungi. *Ann. Rev. Entomol.* 59 467–485. 10.1146/annurev-ento-011613-162054 24160418

[B4] Cerenius L. Lee B. L. Soderhall K. (2008). The proPO-system: pros and cons for its role in invertebrate immunity. *Trends Immunol.* 29 263–271. 10.1016/j.it.2008.02.009 18457993

[B5] Cerenius L. Söderhäll K. (2004). The prophenoloxidase-activating system in invertebrates. *Immunol. Rev.* 198 116–126. 15199959 10.1111/j.0105-2896.2004.00116.x

[B6] Conesa A. Gotz S. Garcia-Gomez J. M. Terol J. Talon M. Robles M. (2005). Blast2GO: a universal tool for annotation, visualization and analysis in functional genomics research. *Bioinformatics* 21 3674–3676. 10.1093/bioinformatics/bti610 16081474

[B7] Fauvarque M. O. Williams M. J. (2011). *Drosophila* cellular immunity: a story of migration and adhesion. *J. Cell Sci.* 124(Pt 9), 1373–1382. 10.1242/jcs.064592 21502134

[B8] Feng H. Chen P. Li G. Qiu F. Guo X. (2012). Diapause induction in *Apolygus lucorum* and *Adelphocoris suturalis* (Hemiptera: Miridae) in northern China. *Environ. Entomol.* 41 1606–1611. 10.1603/EN12099 23321109

[B9] Ferrandon D. Imler J. L. Hetru C. Hoffmann J. A. (2007). The Drosophila systemic immune response: sensing and signalling during bacterial and fungal infections. *Nat. Rev. Immunol.* 7 862–874. 10.1038/nri2194 17948019

[B10] Fu L. Niu B. Zhu Z. Wu S. Li W. (2012). CD-HIT: accelerated for clustering the next-generation sequencing data. *Bioinformatics* 28 3150–3152. 10.1093/bioinformatics/bts565 23060610 PMC3516142

[B11] Gottar M. Gobert V. Matskevich A. A. Reichhart J.-M. Wang C. Butt T. M. (2006). Dual detection of fungal infections in *Drosophila* via recognition of glucans and sensing of virulence factors. *Cell* 127 1425–1437. 10.1016/j.cell.2006.10.046 17190605 PMC1865096

[B12] Gottar M. Gobert V. Michel T. Belvin M. Duyk G. Hoffmann J. A. (2002). The *Drosophila* immune response against Gram-negative bacteria is mediated by a peptidoglycan recognition protein. *Nature* 416 640–644. 11912488 10.1038/nature734

[B13] Grabherr M. G. Haas B. J. Yassour M. Levin J. Z. Thompson D. A. Amit I. (2011). Full-length transcriptome assembly from RNA-Seq data without a reference genome. *Nat. Biotechnol.* 29 644–652. 10.1038/nbt.1883 21572440 PMC3571712

[B14] Hedengren M. Asling B. Dushay M. S. Ando I. Ekengren S. Wihlborg M. (1999). Relish, a central factor in the control of humoral but not cellular immunity in Drosophila. *Mol. Cell* 4 827–837. 10619029 10.1016/s1097-2765(00)80392-5

[B15] Kanehisa M. Goto S. (2000). KEGG: kyoto encyclopedia of genes and genomes. *Nucleic Acids Res.* 28 27–30. 10.1093/nar/28.1.27 10592173 PMC102409

[B16] Kim M. Lee J. H. Lee S. Y. Kim E. Chung J. (2006). Caspar, a suppressor of antibacterial immunity in *Drosophila*. *Proc. Natl. Acad. Sci. U S A.* 103 16358–16363. 17050695 10.1073/pnas.0603238103 PMC1637587

[B17] Lemaitre B. Hoffmann J. (2007). The host defense of *Drosophila melanogaster*. *Ann. Rev. Immunol.* 25 697–743. 10.1146/annurev.immunol.25.022106.141615 17201680

[B18] Lemaitre B. Kromer-Metzger E. Michaut L. Nicolas E. Meister M. Georgel P. (1995). A recessive mutation, immune deficiency (imd), defines two distinct control pathways in the *Drosophila* host defense. *Proc. Natl. Acad. Sci. U S A.* 92 9465–9469. 7568155 10.1073/pnas.92.21.9465 PMC40822

[B19] Lemaitre B. Nicolas E. Michaut L. Reichhart J. M. Hoffmann J. A. (1996). The dorsoventral regulatory gene cassette spätzle/Toll/cactus controls the potent antifungal response in *Drosophila* adults. *Cell* 86 973–983. 8808632 10.1016/s0092-8674(00)80172-5

[B20] Lemaitre B. Reichhart J. M. Hoffmann J. A. (1997). Drosophila host defense: differential induction of antimicrobial peptide genes after infection by various classes of microorganisms. *Proc. Natl. Acad. Sci. U S A.* 94 14614–14619. 9405661 10.1073/pnas.94.26.14614 PMC25070

[B21] Leulier F. Parquet C. Pili-Floury S. Ryu J. H. Caroff M. Lee W. J. (2003). The Drosophila immune system detects bacteria through specific peptidoglycan recognition. *Nat. Immunol.* 4 478–484. 10.1038/ni922 12692550

[B22] Li B. Dewey C. N. (2011). RSEM: accurate transcript quantification from RNA-Seq data with or without a reference genome. *BMC Bioinform.* 12:323. 10.1186/1471-2105-12-323 21816040 PMC3163565

[B23] Li G. Feng H. Chen P. Wu S. Liu B. Qiu F. (2010). Effects of transgenic Bt cotton on the population density, oviposition behavior, development, and reproduction of a nontarget pest, *Adelphocoris suturalis* (Hemiptera: Miridae). *Environ. Entomol.* 39 1378–1387. 10.1603/EN09223 22127190

[B24] Li W. Godzik A. (2006). Cd-hit: a fast program for clustering and comparing large sets of protein or nucleotide sequences. *Bioinformatics* 22 1658–1659. 10.1093/bioinformatics/btl158 16731699

[B25] Liu F. Yang B. Zhang A. Ding D. Wang G. (2019). Plant-Mediated RNAi for controlling *Apolygus lucorum*. *Front. Plant Sci.* 10:64. 10.3389/fpls.2019.00064 30792724 PMC6374644

[B26] Lü J. Yang C. Zhang Y. Pan H. (2018). Selection of reference genes for the normalization of RT-qPCR data in gene expression studies in insects: a systematic review. *Front. Physiol.* 9:1560. 10.3389/fphys.2018.01560 30459641 PMC6232608

[B27] Lu Y. Jiao Z. Li G. Wyckhuys K. A. G. Wu K. (2011). Comparative overwintering host range of three *Adelphocoris species* (Hemiptera: Miridae) in northern China. *Crop Protect.* 30 1455–1460. 10.1016/j.cropro.2011.07.010

[B28] Lu Y. Wu K. (2011). Mirid bugs in China: pest status and management strategies. *Outlooks Pest Manag.* 22 248–252. 10.1564/22dec02 16562962

[B29] Luo J. Li Z. Ma C. Zhang Z. Hull J. J. Lei C. (2017a). Knockdown of a metathoracic scent gland desaturase enhances the production of (E)-4-oxo-2-hexenal and suppresses female sexual attractiveness in the plant bug *Adelphocoris suturalis*. *Insect Mol. Biol.* 26 642–653. 10.1111/imb.12325 28621451

[B30] Luo J. Liang S. Li J. Xu Z. Li L. Zhu B. (2017b). A transgenic strategy for controlling plant bugs (*Adelphocoris suturalis*) through expression of double-stranded RNA homologous to fatty acyl-coenzyme a reductase in cotton. *New Phytol.* 215 1173–1185. 10.1111/nph.14636 28608990

[B31] Luo J. Liu X. Liu L. Zhang P. Chen L. Gao Q. (2014). De novo analysis of the *Adelphocoris suturalis Jakovlev metathoracic* scent glands transcriptome and expression patterns of pheromone biosynthesis-related genes. *Gene* 551 271–278. 10.1016/j.gene.2014.09.004 25194898

[B32] Luo J. Ma C. Li Z. Zhu B. Zhang J. Lei C. (2018). Assessment of suitable reference genes for qRT-PCR analysis in *Adelphocoris suturalis*. *J. Integrat. Agricul.* 17 149–161.

[B33] Mascarin G. M. Jaronski S. T. (2016). The production and uses of *Beauveria bassiana* as a microbial insecticide. *World J. Microbiol. Biotechnol.* 32:177. 10.1007/s11274-016-2131-3 27628337

[B34] Michel T. Reichhart J. M. Hoffmann J. A. Royet J. (2001). *Drosophila* toll is activated by gram-positive bacteria through a circulating peptidoglycan recognition protein. *Nature* 414 756–759. 11742401 10.1038/414756a

[B35] Moret Y. Moreau J. (2012). The immune role of the arthropod exoskeleton. *Adv. Insect Physiol.* 9 200–206.

[B36] Portilla M. Abbas H. K. Accinelli C. Luttrell R. (2019). Laboratory and field investigations on compatibility of *Beauveria bassiana* (Hypocreales: Clavicipitaceae) spores with a sprayable bioplastic formulation for application in the biocontrol of tarnished plant bug in cotton. *J. Econ. Entomol.* 112 549–557. 10.1093/jee/toy382 30561663

[B37] Richman A. M. Bulet P. Hetru C. Barillas-Mury C. Hoffmann J. A. Kafalos F. C. (1996). Inducible immune factors of the vector mosquito *Anopheles gambiae*: biochemical purification of a defensin antibacterial peptide and molecular cloning of preprodefensin cDNA. *Insect Mol. Biol.* 5 203–210. 8799739 10.1111/j.1365-2583.1996.tb00055.x

[B38] Schmittgen T. D. Livak K. J. (2008). Analyzing real-time PCR data by the comparative C(T) method. *Nat. Protoc.* 3 1101–1108. 10.1038/nprot.2008.73 18546601

[B39] Tian C. Tek Tay W. Feng H. Wang Y. Hu Y. Li G. (2015). Characterization of *Adelphocoris suturalis* (Hemiptera: Miridae) transcriptome from different developmental stages. *Sci. Rep.* 5:11042. 10.1038/srep11042 26047353 PMC4457133

[B40] Trapnell C. Williams B. A. Pertea G. Mortazavi A. Kwan G. van Baren M. J. (2010). Transcript assembly and quantification by RNA-Seq reveals unannotated transcripts and isoform switching during cell differentiation. *Nat. Biotechnol.* 28 511–515. 10.1038/nbt.1621 20436464 PMC3146043

[B41] Valero-Jimenez C. A. Wiegers H. Zwaan B. J. Koenraadt C. J. van Kan J. A. (2016). Genes involved in virulence of the entomopathogenic fungus *Beauveria bassiana*. *J. Invertebrate Pathol.* 133 41–49. 10.1016/j.jip.2015.11.011 26628209

[B42] Wang C. Wang S. (2017). Insect pathogenic fungi: genomics, molecular interactions, and genetic improvements. *Ann. Rev. Entomol.* 62 73–90. 10.1146/annurev-ento-031616-035509 27860524

[B43] Wang L. Feng Z. Wang X. Wang X. Zhang X. (2010). DEGseq: an R package for identifying differentially expressed genes from RNA-seq data. *Bioinformatics* 26 136–138. 10.1093/bioinformatics/btp612 19855105

[B44] Wu K. M. Lu Y. H. Feng H. Q. Jiang Y. Y. Zhao J. Z. (2008). Suppression of cotton bollworm in multiple crops in China in areas with Bt toxin-containing cotton. *Science* 321 1676–1678. 10.1126/science.1160550 18801998

[B45] Xiong G. H. Xing L. S. Lin Z. Saha T. T. Wang C. Jiang H. (2015). High throughput profiling of the cotton bollworm Helicoverpa armigera immunotranscriptome during the fungal and bacterial infections. *BMC Genom.* 16:321. 10.1186/s12864-015-1509-1 26001831 PMC4490664

[B46] Xu L. Zhang Y. Zhang S. Deng J. Lu M. Zhang L. (2018). Comparative analysis of the immune system of an invasive bark beetle, *Dendroctonus valens*, infected by an entomopathogenic fungus. *Dev. Comp. Immunol.* 88 65–69. 10.1016/j.dci.2018.07.002 30017857

[B47] Yajuan T. Wu. (2010). Pathogenicity of *Beauveria spp*.strains to three species of mirids, *Apolygus lucorum*, *Adelphocoris suturalis* and *Adelphocoris lineolatus*. *Acta Phytophylacica Sinica* 37 172–176.

[B48] Yang H. F. Pan A. J. Hu L. F. Liu Y. Y. Cheng J. Ye Y. (2017). Galleria mellonella as an in vivo model for assessing the efficacy of antimicrobial agents against *Enterobacter cloacae* infection. *J. Microbiol. Immunol. Infect.* 50 55–61. 10.1016/j.jmii.2014.11.011 25682237

[B49] Zhang W. Chen J. Keyhani N. O. Zhang Z. Li S. Xia Y. (2015). Comparative transcriptomic analysis of immune responses of the migratory locust, Locusta migratoria, to challenge by the fungal insect pathogen, *Metarhizium acridum*. *BMC Genom.* 16:867. 10.1186/s12864-015-2089-9 26503342 PMC4624584

[B50] Zhang W. Meng J. Ning J. Qin P. Zhou J. Zou Z. (2017). Differential immune responses of *Monochamus alternatus* against symbiotic and entomopathogenic fungi. *Sci. China Life Sci.* 60 902–910. 10.1007/s11427-017-9102-y 28762123

[B51] Zhang Z. Luo J. Lu C. Zhao B. Meng J. Chen L. (2011). Evidence of female-produced sex pheromone of *Adelphocoris suturalis* (Hemiptera: Miridae): effect of age and time of day. *J. Econ. Entomol.* 104 1189–1194. 10.1603/ec11006 21882682

[B52] Zou Z. Shin S. W. Alvarez K. S. Bian G. Kokoza V. Raikhel A. S. (2008). Mosquito RUNX4 in the immune regulation of PPO gene expression and its effect on avian malaria parasite infection. *Proc. Natl. Acad. Sci. U S A.* 105 18454–18459. 10.1073/pnas.0804658105 19011100 PMC2587535

[B53] Zou Z. Shin S. W. Alvarez K. S. Kokoza V. Raikhel A. S. (2010). Distinct melanization pathways in the mosquito *Aedes aegypti*. *Immunity* 32 41–53. 10.1016/j.immuni.2009.11.011 20152169

